# Expression of oncogenic HRAS ^G12V^ causes defects in control of cell size in NIH 3T3 cells

**DOI:** 10.17912/micropub.biology.000873

**Published:** 2023-11-02

**Authors:** Jerry T. DeWitt, Michael V. Sharma, Douglas R. Kellogg

**Affiliations:** 1 Molecular, Cell, and Developmental Biology, University of California, Santa Cruz, Santa Cruz, California, United States

## Abstract

Severe defects in control of cell size are closely associated with cancer. However, the mechanisms that drive cell size defects in cancer remain unknown and it is unclear whether they are a direct consequence of signals from primary oncogenic drivers or a secondary consequence of mutations that accumulate during evolution of cancer cells. Here, we report that expression of oncogenic HRAS ^G12V^ is sufficient to cause cell size defects in NIH 3T3 cells, which suggests that the cell size defects of cancer cells are a direct consequence of primary oncogenic drivers.

**Figure 1. Expression of oncogenic HRAS G12V causes an increase in cell size in NIH 3T3 cells:**
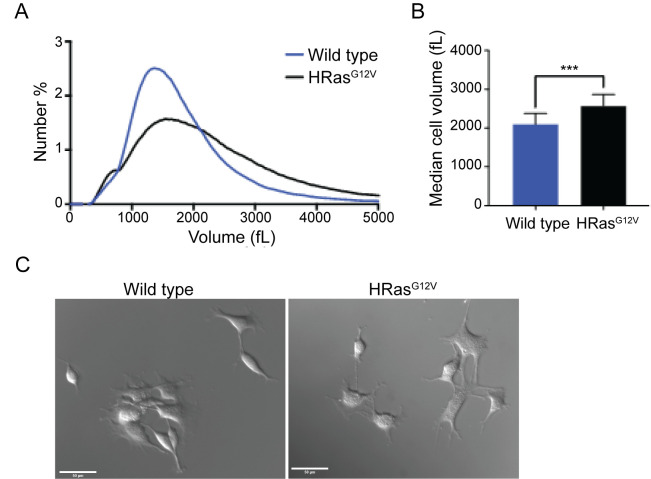
Figure 1 A) Wild type and HRAS ^G12V^ NIH 3T3 cells were plated and grown for 48 hours in high glucose DMEM supplemented with 10% FCS and 1% Pen/Strep and cell size was measured using a Coulter Counter. Size plots represent the averages of 5 biological replicates. B) Histograms representing the mean cell size (MCV) for Wild type and HRAS ^G12V^ NIH 3T3 cells. Error bars represent SEM for 5 biological replicates. *** indicates a p value < 0.001 by a paired t-test. C) Images of wild type and HRAS ^G12V^ NIH 3T3 cells.

## Description

Many different kinds of cancer cells show aberrant cell size, as well as greater heterogeneity in cell size and severely perturbed cytoplasmic to nuclear volume ratios. (Asa, 2019; Asadullah et al., 2021; Hoda et al., 2018; Gothwal et al., 2021; Sandlin et al., 2022) . These defects have long been used to help identify and evaluate cancer cells in clinical tests. For example, the Pap smear test and the Gleason grading system identify cancer cells via their aberrant size and shape, and increased severity of these defects is associated with a poor prognosis (Brimo et al., 2013; Epstein et al., 2005; Chen and Zhou, 2016; Trpkov, 2015; Sandlin et al., 2022) . However, the molecular mechanisms that drive cell size defects in cancer are unknown. Discovery of these mechanisms could yield new insights into the aberrant biology of cancer cells that could be targeted with new therapies.

The transformation of a normal cell into a cancer cell is a multi-step process that requires mutations in numerous genes. Although many cancers show defects in cell size, it is unclear whether the defects are a direct consequence of primary oncogenic signals or a secondary consequence of mutations that accumulate as cells evolve and adapt to oncogenic signals. No previous studies have directly tested the effects of a primary oncogenic driver on cell size in the absence of the many additional mutations that accumulate during evolution of cancer cells. Here, we tested the effects of the HRAS ^G12V^ oncogene in NIH 3T3 cells. The Ras family of GTPases are conserved from yeast to mammals and function as master regulators of growth, proliferation, metabolism and survival. Oncogenic forms of Ras carry mutations that block GTP hydrolysis, thereby locking Ras into a GTP-bound hyperactive state that promotes aberrant proliferation. Ras genes are amongst the most frequently mutated oncogenic drivers; it is thought that between 25-30% of all human cancers have oncogenic mutations in one or more Ras genes. Recent work in budding yeast found that a hyperactive allele of Ras that is analogous to oncogenic Ras in mammalian cells is sufficient to drive an increase in cell size (DeWitt et al., 2023) . Another recent study found that KRAS-driven lung adenocarcinoma tumor samples have dramatically increased cell size, but did not examine the effects of KRAS in the absence of additional passenger mutations present in tumor cells (Sandlin et al., 2022) . Together these data suggest that aberrant Ras signaling may influence cell size control in both yeast and mammals.

To test whether expression of HRAS ^G12V^ is sufficient to cause cell size defects, we utilized a previously generated NIH 3T3 cell line that expresses HRAS ^G12V^ , as well as an isogenic control cell line (Vichas et al., 2021) . A Coulter Counter was used to measure the size of the HRAS ^G12V^ cells and the isogenic control cells. This showed that HRas ^G12V^ causes a substantial increase in cell size across the cell population ( Figure 1A ), and an approximately 500 fL increase in average cell size ( Figure 1B ). These data show that expression of a major oncogenic driver is sufficient to cause substantial cell size defects. Images of the cells are shown in Figure 1C .

The mechanism by which HRAS ^G12V^ influences cell size is unknown. In budding yeast, hyperactive Ras causes a delay in cell cycle entry (DeWitt et al., 2023) . Cell growth continues during the delay, which causes cells to enter the cell cycle at a larger size. Therefore, in mammalian cells it is possible that HRAS ^G12V^ influences cell size by causing a cell cycle delay that leads to a longer interval of growth. Alternatively, HRAS ^G12V^ could influence signals that set the threshold amount of growth required for cell cycle progression. Further work is needed to determine how oncogenic signals cause perturbations of cell size in mammalian cells.

## Methods

Wild type and HRAS ^G12V^ NIH 3T3 cells were previously generated (Vichas et al., 2021) . NIH 3T3 Cells were cultured at 37ºC/5% CO _2_ in DMEM high glucose medium with L-Glutamine, no Sodium PyruvateEM (Cytiva #SH30022.01) supplemented with 10% bovine calf serum (Sigma #12133C) and 1% penicillin + 1% streptomycin (Thermo #15-140-122). Testing for mycoplasma contamination was carried out via PCR using the following oligos:

Forward primers: *CGCCTGAGTAGTACGTTCGC, CGCCTGAGTAGTACGTACGC, TGCCTGAGTAGTACATTCGC, TGCCTGGGTAGTACATTCGC, CGCCTGGGTAGTACATTCGC, CGCCTGAGTAGTATGCTCGC.*

Reverse primers: *GCGGTGTGTACAAGACCCGA, GCGGTGTGTACAAAACCCGA, GCGGTGTGTACAAACCCCGA* .

For analysis by Coulter Counter, cells were seeded at ~20-30% confluence and grown for 2 days at 37ºC with 5% CO2 before harvesting. Cells were never cultured beyond ~90% confluence and were analyzed at the same passage. To harvest, cells were trypsinized and then quenched with complete DMEM media containing 10% bovine calf serum prior to centrifugation for 5 minutes at 2300 RPM. Cells were fixed with 3.7% formaldehyde for 30 min and were then washed with PBS + 0.02% Tween-20 + 0.02% sodium azide. Cells were then filtered through a 40 µM nylon membrane (Corning ref.# 352340) to eliminate clumped cells before measuring cell size using a Z2 Coulter Counter with AccuComp v3.01a software. The data in Figure 1 represent the average of 5 independent biological replicates, in which each biological replicate is the average of multiple technical replicates. All biological replicates consistently showed that HRAS ^G12V^ cells are larger than the wild type control cells.

Figures and Statistical analysis

Figures were assembled using Affinity Designer 2. A paired t-test was performed on the mean cell size for 5 biological replicates using Prism 9 (Grahpad) and p values are described in the figure legend.

## References

[R1] Asa Sylvia L. (2019). The Current Histologic Classification of Thyroid Cancer. Endocrinology and Metabolism Clinics of North America.

[R2] Asadullah, Kumar S, Saxena N, Sarkar M, Barai A, Sen S (2021). Combined heterogeneity in cell size and deformability promotes cancer invasiveness.. J Cell Sci.

[R3] Brimo F, Montironi R, Egevad L, Erbersdobler A, Lin DW, Nelson JB, Rubin MA, van der Kwast T, Amin M, Epstein JI (2012). Contemporary grading for prostate cancer: implications for patient care.. Eur Urol.

[R4] Chen N, Zhou Q (2016). The evolving Gleason grading system.. Chin J Cancer Res.

[R5] DeWitt JT, Chinwuba JC, Kellogg DR (2023). Hyperactive Ras disrupts cell size control and a key step in cell cycle entry in budding yeast.. Genetics.

[R6] Epstein JI, Allsbrook WC Jr, Amin MB, Egevad LL, ISUP Grading Committee (2005). The 2005 International Society of Urological Pathology (ISUP) Consensus Conference on Gleason Grading of Prostatic Carcinoma.. Am J Surg Pathol.

[R7] Gothwal M, Nalwa A, Singh P, Yadav G, Bhati M, Samriya N (2020). Role of Cervical Cancer Biomarkers p16 and Ki67 in Abnormal Cervical Cytological Smear.. J Obstet Gynaecol India.

[R8] Hoda RS, Lu R, Arpin RN 3rd, Rosenbaum MW, Pitman MB (2018). Risk of malignancy in pancreatic cysts with cytology of high-grade epithelial atypia.. Cancer Cytopathol.

[R9] Sandlin CW, Gu S, Xu J, Deshpande C, Feldman MD, Good MC (2022). Epithelial cell size dysregulation in human lung adenocarcinoma.. PLoS One.

[R10] Trpkov Kiril (2015). Contemporary Gleason Grading System. Genitourinary Pathology.

[R11] Vichas A, Riley AK, Nkinsi NT, Kamlapurkar S, Parrish PCR, Lo A, Duke F, Chen J, Fung I, Watson J, Rees M, Gabel AM, Thomas JD, Bradley RK, Lee JK, Hatch EM, Baine MK, Rekhtman N, Ladanyi M, Piccioni F, Berger AH (2021). Integrative oncogene-dependency mapping identifies RIT1 vulnerabilities and synergies in lung cancer.. Nat Commun.

